# Comparative Evaluation of Apically Extruded Debris during Pulpectomy Procedure in Primary Molar Teeth Using Two Different Rotary Systems and Hand Files: An In Vitro Study

**DOI:** 10.1155/2022/9433225

**Published:** 2022-06-06

**Authors:** Komal Nanavati, Farhin Katge, Manohar Poojari, Shilpa Shetty, Aishwarya Kamble

**Affiliations:** Department of Pediatric and Preventive Dentistry, T.P.C.T's Terna Dental College and Hospital, Sector-22, Nerul West, Navi Mumbai 4007062, Maharashtra, India

## Abstract

**Background:**

Successful outcome of endodontic treatment depends on chemomechanical preparation of the tooth. However, during canal preparation, debris may extrude through the apical foramen causing postoperative complications. The aim of this study is to evaluate and compare the amount of apically extruded debris during the pulpectomy procedure in primary molar teeth using two different rotary files and hand files.

**Materials and Methods:**

Distal roots of sixty extracted primary molars were divided into three groups: group A: Prime Pedo™ pediatric rotary files, group B: DXL-Pro Pedo™ pediatric rotary files, and group C: hand files. Myers and Montgomery experimental model was used. Preweighed Eppendorf tubes were used to collect apically extruded debris. The tubes were then placed in an incubator at 70°C for 5 days. The weight of the debris attained was determined. The data were analyzed using ANOVA and post hoc Bonferroni test.

**Results:**

Hand files produced more apical debris extrusion (*P* < 0.05). Prime Pedo™ pediatric rotary files produced the least debris (*P* < 0.05).

**Conclusion:**

Prime Pedo™ and DXL-Pro Pedo™ pediatric rotary files extruded significantly lower amount of debris apically during pulpectomy as compared to hand files. Rotary files can be considered an alternative to hand files in pediatric endodontics.

## 1. Introduction

Primary teeth are fundamental for the proper growth and development of jaws and muscles in children. They aid in maintaining arch integrity, functioning of the masticatory system, esthetic appearance, and phonation and act as a guide to the erupting permanent teeth [1]. Premature loss of primary teeth may cause the development of deleterious habits, malocclusion, and change in the path of eruption of permanent teeth which affects the overall growth and esthetics of the child [2]. Periapical or dentoalveolar infection is a major contributing factor for premature loss of primary teeth; therefore, conservative treatment is recommended wherein pulp has the potential to recover from insult [3]. Endodontic intervention is indicated in teeth with irreversible pulpitis and signs of chronic inflammation or necrosis [4]. Pulpectomy helps retain primary teeth in a functional state till the eruption of permanent teeth [5]. Sometimes pediatric endodontic treatment is challenging due to anatomic complexities, physiologic resorption, and also behavioral considerations [6].

During canal preparation, the most commonly encountered setback is that dental shavings, necrotic debris, irrigating solution, and microorganisms may be accidentally pushed into the periapical area. These irritants may result in detrimental outcomes like postoperative pain inflammation and delayed periapical healing [1]. Since primary teeth display various anatomic variations with respect to size, internal-external morphology, and changes in the root length due to physiologic root resorption, there is greater risk for debris extrusion [7].

Therefore, better instrumentation techniques need to be assessed, with the goal of instrumentation directed towards reducing the quantity of debris extruded apically, thus consequently reducing the postoperative symptoms [7]. Limited data are available concerning the use of NiTi rotary files used for deciduous teeth and apical extrusion of debris. Thus, the aim of the present study was to evaluate and compare the amount of apically extruded debris during the pulpectomy procedure in extracted primary molar teeth using two different rotary files, i.e., Prime Pedo™ pediatric rotary files and DXL-Pro Pedo™ pediatric rotary files, and hand files.

## 2. Material and Methods

This study was carried out to evaluate and compare apically extruded debris using two different pediatric rotary files and H-files during the pulpectomy procedure in primary molar teeth. The study was approved by the Institutional Review Board (IRB) with reference number IEC/TDC/19/2018. Sample size was determined in concordance with results obtained from a previous study by Buldur et al. [7] through *G*^*∗*^ power software (version 3.0.10). Sixty mandibular primary molars extracted because of periapical pathology, orthodontic purpose, or nonrestorability of coronal structure with at least two-third root length present were selected. Teeth with internal or external pathological root resorption, root caries, fractures, or cracks were excluded. Each selected tooth was submitted to scaling and root planing using an ultrasonic device to eliminate vestigial organic tissue. Intraoral periapical radiographs (IOPA) were taken buccolingually and mesiodistally to confirm single canal in the distal root and exclude any aberrant canal morphology. Teeth were then stored in distilled water at room temperature until further use. Teeth were sectioned longitudinally at furcation with a low-speed diamond disc under water cooling. Distal root of mandibular primary molars was selected, and mesial roots were discarded. Access cavity was prepared using high-speed airotor handpiece with water cooling and BR-46 diamond bur (Mani Inc., Japan). Canal patency and working length were established with a 21 mm, number 10 K-file (Mani Inc., Japan). The file was inserted into the root canal till its tip was visible at the apical foramen. The working length was calculated by subtracting 1 mm from this measurement [7]. Each canal was then negotiated with number 15 K-file up to the working length.

All tooth segments were attached to a preweighted Eppendorf tube. The preintervention weight of each tube was recorded using analytical microbalance 10-6 g precision. Each tube was weighed 3 times without its lid, and mean value was calculated. Following this, a window was created using a heated ball burnisher on the lid of the tube for attaching the distal root segment. The extruded debris was collected in the tube during canal preparation of the suspended root segment.

**Figure 1:**
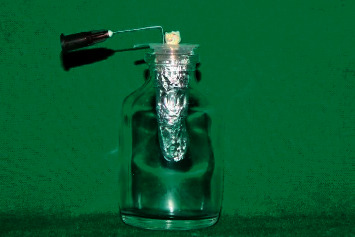
Myers and Montgomery apparatus.

Modified Myers and Montgomery [8] apparatus was used, and the lid of the Eppendorf tube for each sample was fitted with a 27 G needle. It served as a drainage cannula to equalizing air pressure inside and outside the Eppendorf tube. The entire assembly was covered using aluminium foil and fitted into a glass vial. This prevented the operator from viewing debris extrusion during instrumentation (Figure 1) [9].

The Eppendorf tubes with teeth were randomly divided into three groups (*n* = 20):

  Group A—Prime Pedo™ pediatric rotary files
  Group B—DXL-Pro Pedo™ pediatric rotary files
  Group C—H-files.

### 2.1. Canal Preparation for Group A and Group B

**Figure 2:**
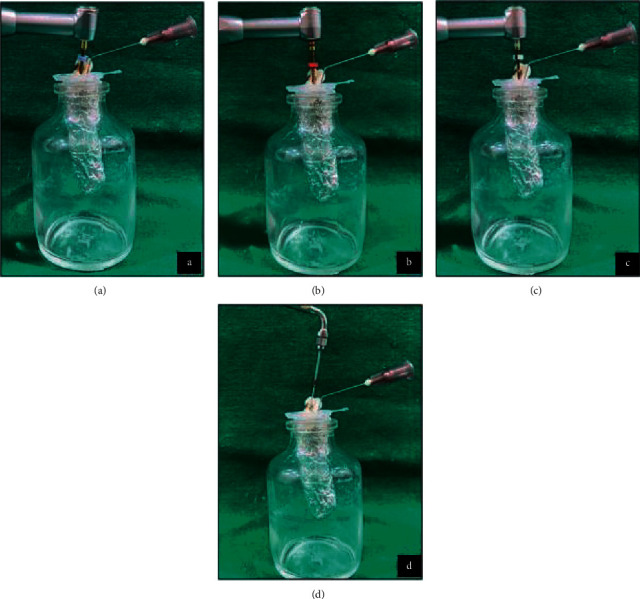
Procedure for group.

Prime Pedo™ pediatric rotary file system consists of starter (8% taper, 16 mm), *P*1 (#15, 6% taper, 18 mm), *P*2 (#25, 6% taper, 18 mm), and endosonic file (2% taper, 18 mm). The starter file was used for orifice enlargement. The coronal and middle third canal preparation was carried out using *P*1 and *P*2. Endosonic file was used for apical preparation. The 2% taper of endosonic file assists in preparing the apex conservatively (Figure 2).

DXL-Pro Pedo™ pediatric rotary files have three files: #30, #20, and #25. The file with size 30 (8% taper, 16 mm length) was used for orifice enlargement. This was followed by the use of file size 20 (6% taper, 18 mm length) and file size 25 (6% taper, 18 mm length).

Prime Pedo™ pediatric rotary files and DXL-Pro Pedo™ pediatric rotary files have a convex triangular cross section and guiding noncutting tip. Crown-down method of instrumentation was used. Instrumentation was done using Endo- Mate DT (NSK, Japan) handpiece at speed of 350 rpm and a torque of 2.4 N/cm as per the manufacturer's instructions. Irrigation was carried out using distilled water.

### 2.2. Canal Preparation for Group C

In group C, H-files were used employing in and out filing motion. Reference [10] files ranging from size 15 to 30 were used to carry out circumferential filing in an ascending order. Irrigation was carried out using distilled water.

**Figure 3:**
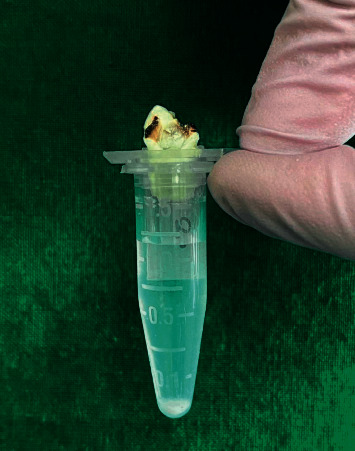
Collected debris in Eppendorf tube.

After completion of canal preparation, the lid with needle and distal root was detached from the tube and the debris adhering to root surface was attained by washing the root with 1 ml distilled water (Figure 3)

The tube was then stored in an incubator at 7°Celsius for 5 days to let the distilled water evaporate. Instrumentation in all three groups was performed by a single operator to facilitate consistent instrumentation protocols. A blinded second examiner assessed the amount of extruded debris.

**Figure 4:**
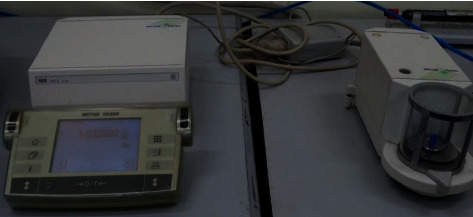
Postintervention weighing of Eppendorf tube in microbalance.

Each tube containing extruded debris was weighed without its lid using the analytical weight microbalance 10^−6^ g three times and the mean value was recorded. This was the final postintervention weight of the tube (Figure 4).

The dry weight of the extruded debris was calculated by subtracting the weight of the empty tube from that of the tube containing debris.

Statistical analysis was performed using windows-based SPSS (SPSS Inc., Chicago, IL, USA) statistics software version 18. Data collected were analyzed statistically using the quantitative number of extruded debris for each group it compared for differences using one-way ANOVA test followed by intergroup comparison using the Bonferroni test.

All testing were done using two-sided tests alpha 0.05. Thus, the criteria for rejecting the null hypothesis was “*P*” value of <0.05.

## 3. Results

### 3.1. Descriptive Statistics

The mean weight and standard deviation for group A were 0.0009614 ± 0.000500 g, group B were 0.0011119 ± 0.000705 g, and group C were 0.001890 ± 0.000850 g. The mean dry weight of extruded debris was used as a comparison between the three groups.

**Figure 5:**
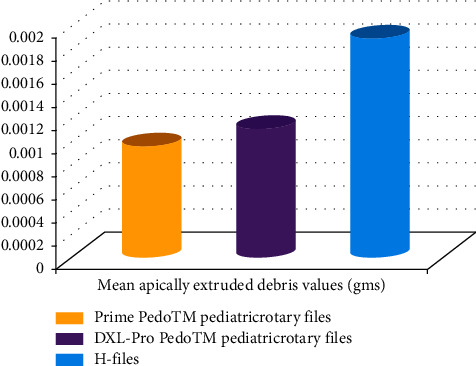
Mean weight of apically extruded of debris during pulpectomy procedure in groups A, B, and C.

The data are illustrated for all three groups in the form of a colored bar graph (Figure 5).

Table 1 demonstrated mean weight, standard deviation, standard error, and higher and lower value of confidence interval at 95% of apically extruded debris carried out using one-way ANOVA. The *P* value of the ANOVA test is 0.001 (*P* < 0.005). Therefore, significant difference in apically extruded debris was observed between all three groups. Group A showed the least weight of extruded debris followed by group B and group C, respectively. Group C showed the greatest amount of apical extrusion of debris.

### 3.2. Intergroup Comparison

Intergroup comparison was carried out using post hoc Bonferroni test for the weight of apically extruded debris with each group. This was carried out to test the null hypothesis.  Table 2 depicts the results of the intergroup comparison. Group A showed significantly less apical extrusion of debris as compared to group C (*P*=0.001). Group B also showed significantly less apical extrusion of debris as compared to group C (*P*=0.003). There was no statistically significant difference between the quantity of debris extruded in group A and group B (*P*=1.00) (Table 2).

## 4. Discussion

The clinical success of pulpectomy lies in various factors such as chemomechanical preparation, apical and coronal seal, restorative material, number of visits, and obturating material [11]. Optimal quality of obturation is achieved when a good chemomechanical preparation is executed so as to obtain uniform and tapered canals [12]. Conventional endodontic treatment for primary teeth is carried out with K-files and H-files. H-files are favored in primary teeth since they enter canals readily with minimum resistance. H-files remove hard tissue on withdrawal, thus preventing infected material from being pushed out of the apex [13]. Although hand instrumentation is considered to be the most satisfactory method for canal shaping and debridement, it can sometimes result in iatrogenic errors such as perforation and ledge formation and is generally time-consuming [14]. Therefore, new innovations and developments over the past decades have led to an exemplary shift in the field of dentistry. In the field of pediatric endodontics, rotary NiTi files were first employed by Barr et al. in 2000. Rotary instrumentation in primary teeth was predominantly advocated due to inherent flexibility and superior resistance to torsional fracture of NiTi files [15]. This makes them more flexible as compared to stainless steel files and helps preserve the original anatomy of curved canals in primary molars [16]. The mechanical instrumentation of canals is of reduced intensity with hand files. The instrumentation must taper the canal and make it easily cleanable; therefore, the instruments must be heat treated and martensitic [17].

Postoperative pain and swelling are common undesirable complications encountered following instrumentation during endodontic treatment in primary and permanent teeth. Rendering superior treatment with the least postoperative pain should be the goal for successful endodontic treatment. Seltzer and Naidorf have promulgated apical extrusion of debris as the most prevalent cause of postoperative pain [18]. Extrusion of debris from the root canals during instrumentation was first documented by Chapman et al. [19]. This extruded debris usually contains pulp remnants, dentine shavings, necrotic tissues, and irrigation solution with microorganisms. This may cause pain, periradicular inflammation with delayed healing, and swelling and could lead to damage to developing permanent tooth [1].

Primary teeth may exhibit more apical extrusion of debris due to wider apical diameter and physiologic root resorption [20]. Thus, considering the clinical relevance, it is important to assess new file systems which can be used to minimize debris extrusion. In most studies, rotary instrumentation in primary teeth was carried out using rotary files devised particularly for permanent teeth. Pediatric patients have lesser mouth opening as compared to adults making it difficult to work with rotary files used for permanent teeth. Additionally, primary teeth have complex thin ribbon-shaped canal anatomy which are challenging to negotiate with rotary files utilized in permanent teeth [21]. Recently, various pediatric rotary files have been introduced in the market to overcome the difficulties caused due to using rotary files for permanent teeth. There is limited literature on apical extrusion of debris using pediatric rotary files in primary teeth. Hand files serve as the standard instrumentation technique for pulpectomy [22]. Hence, in the present study, apical extrusion of debris was evaluated using Prime Pedo™ and DXL-Pro Pedo™ pediatric rotary files compared with H-files during pulpectomy procedure. Prime Pedo™ and DXL-Pro Pedo™ were used as they are designed for primary teeth and are easily available.

Resin block models have been used in previous studies to standardize the shape, size, taper, and curvature of root canals [23]. Ruiz-Hubard et al. [24] used acrylic models for standardization, but they lack pulp tissue, three-dimensional curves, canal irregularities, and natural apical constriction. Kum et al. [25] stated another disadvantage of simulated root canals could be the heat generated during rotary instrumentation as it may adversely affect the results by softening the models. Therefore, extracted human primary mandibular molars were selected in the present study to replicate in vivo situations. Distal roots with single canal and at least 2/3rd root length in primary mandibular molars were selected for standardization and to overcome morphological variations. Radiographs were taken to ensure that sample teeth had single canals and maintain uniformity between the three groups. However, as stated by Koçak et al., no distinction was made between first and second mandibular primary molars, and this was done to maintain some degree of anatomical discrepancy among the specimens [26]. The teeth were stored in distilled water to prevent dehydration.

Myers and Montgomery method has been extensively used for collecting, weighing, and evaluating debris extruded via the apical foramen in different studies [27]. Eppendorf tubes containing specimen were covered in aluminium foil and placed in glass vials. This allowed visualization of only canal entrance during instrumentation. This was done to prevent the operator from influencing the results [28]. A single operator carried out instrumentation in all the groups to avoid interoperator variability or bias.

For each sample, the working length was kept 1 mm short of the apex. Previous studies have shown that increasing the apical limit of preparation may lead to greater quantity of extruded debris [29]. Keeping the working length 1 mm short of apex improves postoperative recuperation [8].

The present study demonstrated that all three file systems cause apical extrusion of debris. Prime Pedo™ pediatric rotary files caused least debris extrusion followed by DXL-Pro Pedo™ pediatric rotary files and H-files. A statistically significant difference was observed between Prime Pedo™ pediatric rotary files and H-files as well as DXL-Pro Pedo™ pediatric rotary files and H-files, respectively. Based on the results, the null hypothesis was rejected as the systems differed statistically in the quantity of extruded debris after instrumentation.

In group A, mean score of apical extrusion of debris was 0.000961 g. In group B, mean score of apical extrusion of debris was 0.001111 g. In group C, H-files displayed the highest mean weight of 0.001890 g for apical extrusion of debris. In the present study, instrumentation using hand files produced the largest amount of apical extrusion of debris followed by DXL-Pro Pedo™ pediatric rotary files and Prime Pedo™ pediatric rotary files, respectively. This finding may be attributed to the difference in motion kinematics and file design among two different rotary files as compared to hand files [30]. Some studies have correlated the quantity of extruded debris with cross section and taper [31].

Prime Pedo™ and DXL-Pro Pedo™ pediatric rotary files possess controlled memory and higher fatigue resistance. These properties reduce fracture of rotary files in curved canals of primary molars. Both file systems are heat treated and therefore are less liable to deformation and follow the tortuous root canal system. Their high flexibility prevents accidental file breakage [32]. In this study, lower debris extrusion with rotary files may be due to the triangular cross section and positive rake angle of Prime Pedo™ and DXL-Pro Pedo™ pediatric rotary files. Triangular cross section diminishes contact areas between file and dentin; thus, there is less binding of the file to canal wall. The reduced contact results reduction in the dentinal shavings and more space around the file to accommodate the debris; this decreases inadvertent extrusion of debris apically [33].

H-files are manufactured from round stainless steel wire by machine grinding in the form of a sequence of intersecting cones. This design creates sharp edges at the base of each cone that cuts the tooth structure only when pulled. They have a tear drop-shaped cross section. Higher chances of file separation occur if used in reaming action as the junction between two cones of the instrument is fragile. Therefore, H-files should be used in filing action only [13]. Various studies have concluded that it is time-consuming to prepare a thin curved canal with stainless steel files and requires the restriction of apical enlargement to relatively small sizes [34]. Stainless steel files are rigid and relatively inflexible, leading to difficulty in following natural anatomy of primary molar canals. This gives rise to inadequate instrumentation and reduces the effectiveness of irrigation [35].

Studies have demonstrated that extruded debris was greater with push-pull instrumentation as compared to rotary instrumentation [2, 8]. In groups A and B, the files consisted of greater taper and were used in crown-down technique of canal preparation. The use of greater tapers allows more apical deposition of the irrigant, facilitating a thorough removal of pulp tissue, bacteria, and necrotic debris [36]. Coronal flaring tends to direct debris towards the orifice rather than pushing it apically. The gradual progress of instruments from cervical to apical third using in-out movements and minor taper size difference among the instruments used in group C may be the reason for greater extrusion of debris [37].

Distilled water was used in the present study for irrigation. The amount was standardized to 1 ml for all three groups. To avoid error in results due to deposition of sodium crystals on evaporation of sodium hypochlorite, it was not used for irrigating canals [38]. All feasible measures were taken to prevent the bias.

Topçuoğlu et al. [39] assessed the amount of apically extruded debris using Revo-S, Mtwo, ProTaper Next, and hand files in primary molars with at least two-thirds root length. They concluded that increased extrusion of debris during instrumentation with hand instrumentation can be attributed to the piston-like motion. Rotary instruments direct the debris coronally as compared to hand instruments, thus reducing apically extruded debris. Ferraz et al. [40] reported less extrusion of debris with Profile system when compared to manual technique. Asif et al. [2] evaluated apical extrusion of debris in primary anterior teeth during root canal preparation. They used hand files, rotary ProTaper files, and rotary Kedo-S pediatric rotary files. They concluded that instrumentation using Kedo-S pediatric rotary files caused statistically less significant extrusion of debris as compared to instrumentation with ProTaper and hand files. Preethy et al. [37] compared the quantity of debris extruded apically in primary teeth using hand files, K3, and Kedo-S rotary files. They found a statistically significant difference between hand files, Kedo-S, and K3 files. Maximum debris extrusion was seen with hand files as compared to K3 and Kedo-S files. Buldur et al. [7] compared the quantity of debris extruded apically using ProTaper, ProTaper Next, SAF, and hand files. The authors found that ProTaper Next and SAF showed significantly less apically extruded debris as compared to ProTaper and hand files. Madalena et al. [41] compared the quantity of extruded debris in primary molars using manual instrumentation and WaveOne system. They found that WaveOne system presented lower extruded debris as compared to manual technique, but the difference was not statistically significant.

Thus, the results of the present study are in concordance with various other studies depicting all instruments used during root canal preparation produce apically extruded debris, but instrumentation with rotary NiTi files causes lesser apical extrusion of debris as compared to manual instrumentation.

A limitation in the present study is the buccolingual sectioning of crown for standardization as this is not representative of the clinical scenario. The quantity of extruded debris may be affected by the absence of clinical crown. Another limitation is the absence of physical backpressure provided by the periapical tissues. Periapical tissues resist the extrusion of debris and irrigant from the canals and may affect the quantity of debris extruded periapically [2]. However, this condition was consistent among the three groups. Some studies have used floral foam and agar gel to simulate periapical resistance [42]. However, this type of physical barrier resembling bone or periodontal ligament could retain debris that would otherwise be extruded, thus compromising the reliability of the results [43]. Therefore, in the present study, no attempt was made to simulate periapical tissue resistance.

It is not possible to evaluate apical extrusion of debris in vivo; therefore, clinicians should rely on the techniques that promote lesser amount of debris extrusion in periapical area. More research is needed in this field to evaluate the quantity of extruded debris and its ill effects using different pediatric rotary files.

## 5. Conclusion

Prime Pedo™ and DXL-Pro Pedo™ pediatric rotary files extruded significantly lower amount of debris apically during pulpectomy procedure in primary molar teeth as compared to hand files. Rotary files can be considered better to hand files as they significantly reduce the extrusion of debris, thus reducing postoperative events followed by pulpectomy procedure.

## Figures and Tables

**Table 1:** Mean, standard deviation, standard error, confidence interval, and *P* value for weight of extruded debris in groups A, B, and C, respectively.

Group	*n*	Mean	Standard deviation	Standard error	95% confidence interval for mean		*P* value
					Lower bound	Upper bound	
Group A	20	0.0009614	0.000500	0.000111	0.000727	0.001195	**0.001** ^*∗*^
Group B	20	0.0011119	0.000705	0.000157	0.000781	0.001442	
Group C	20	0.0018902	0.000850	0.000190	0.001492	0.002288	

*Note*. ^*∗*^Level of significance set at *P* < 0.005. The bold value suggests that the result was significant between the three groups. It suggests that the result was significant as *p* value is less than 0.005.

**Table 2:** Intergroup comparison using post hoc Bonferroni test.

Groups	Groups	Mean difference	Std. error	*P* value	95% confidence interval	
					Lower bound	Upper bound
A	B	−0.00015050	0.00022144	1.000	−0.0006967	0.0003957
	C	**−0.00092875** ^*∗*^	0.00022144	**0.001** ^*∗*^	−0.0014750	−0.0003825

B	A	0.00015050	0.00022144	1.000	−0.0003957	0.0006967
	C	**−0.00077825** ^*∗*^	0.00022144	**0.003** ^*∗*^	−0.0013245	−0.0002320

C	A	**0.00092875** ^*∗*^	0.00022144	**0.001** ^*∗*^	0.0003825	0.0014750
	B	**0.00077825** ^*∗*^	0.00022144	**0.003** ^*∗*^	0.0002320	0.0013245

*Note*. ^*∗*^Level of significance set at *P* < 0.005. The bold values show a significant difference between the groups.

## Data Availability

The data supporting the findings of the study are available from the corresponding author upon reasonable request.
